# Assembling non-ferromagnetic materials to ferromagnetic architectures using metal-semiconductor interfaces

**DOI:** 10.1038/srep34404

**Published:** 2016-09-29

**Authors:** Ji Ma, Chunting Liu, Kezheng Chen

**Affiliations:** 1 Lab of Functional and Biomedical Nanomaterials, College of Materials Science and Engineering, Qingdao University of Science and Technology, Qingdao 266042, China

## Abstract

In this work, a facile and versatile solution route was used to fabricate room-temperature ferromagnetic fish bone-like, pteridophyte-like, poplar flower-like, cotton-like Cu@Cu_2_O architectures and golfball-like Cu@ZnO architecture. The ferromagnetic origins in these architectures were found to be around metal-semiconductor interfaces and defects, and the root cause for their ferromagnetism lay in charge transfer processes from metal Cu to semiconductors Cu_2_O and ZnO. Owing to different metallization at their interfaces, these architectures exhibited different ferromagnetic behaviors, including coercivity, saturation magnetization as well as magnetic interactions.

Interest in multifunctional materials with the spin degree of freedom has been steadily increasing in recent years catalyzed by their rapidly expanding applications, ranging from spintronics to quantum technologies^1^,^2^,^3^,^4^,^5^,^6^. Strategies employed to establish magnetic ordering in these materials typically rely on doping magnetic transition metal elements, rare earth and heavy elements with a large atomic moment. This is especially the case for diluted magnetic semiconductors (DMSs), which have received extensive attention ever since the ferromagnetism was observed in (Ga, Mn)As^7^,^8^,^9^,^10^. Nowadays, unremitting efforts have been made to obtain room-temperature ferromagnetism (RTF) in semiconductors doped with various ferromagnetic transition metal elements (e.g., Mn^11^, Cr^12^, Fe^13^, Co^14^ and Ni^15^). A common drawback to these studies is that the dopant elements can segregate to form precipitates or clusters that are actually responsible for the ferromagnetic properties. Such effect must be examined very carefully before the usefulness of these DMSs for spintronics applications can be evaluated^16^. In addition, the fluctuations of growth conditions may also result in undesirable changes of the physical properties of these DMSs obtained by different groups. To tackle these issues, development of DMSs doped by non-ferromagnetic elements (e.g., Cu^17^,^18^,^19^,^20^,^21^, Al^22^, Li^23^, Sc, Ti, V, Cr, C, N^24^ etc.) becomes a thriving research topic in recent years. These materials, exhibiting signs of ferromagnetic behavior, are free of ferromagnetic precipitates, and hence they are considered to be unambiguous DMSs. Unfortunately, their ferromagnetic properties are strongly dependent on their usually tedious fabrication procedures^23^.

Recently, RTF is also observed in numerous undoped oxides such as ZnO, TiO_2_, In_2_O_3_, HfO_2_^25^,^26^,^27^ and CuO^28^, in which defects may play an important role in their magnetic behaviors. Therefore, how to control and engineer defects becomes a very interesting and challenging issue. Along this line, we propose a versatile solution route, rather than traditional solid-phase route, through which non-ferromagnetic components can be self-assembled to ferromagnetic architectures by tailoring metal-semiconductor interfaces. This new route, discarding traditional doping concept, is found to be practicable not only for solvothermal methods, but also for thermal decomposition treatment in fabrication. In this work, we choose copper as the main block of various architectures, since it is an essential ingredient in electronics and spintronics applications. Two other semiconductors Cu_2_O and ZnO are separately incorporated with copper to form fish bone-like, pteridophyte-like, poplar flower-like, cotton-like and golfball-like Cu-based architectures. All of these architectures are found to be ferromagnetic at room temperature due to their tailored Cu-Cu_2_O and Cu-ZnO interfaces.

## Results and Discussion

**Figure 1:**
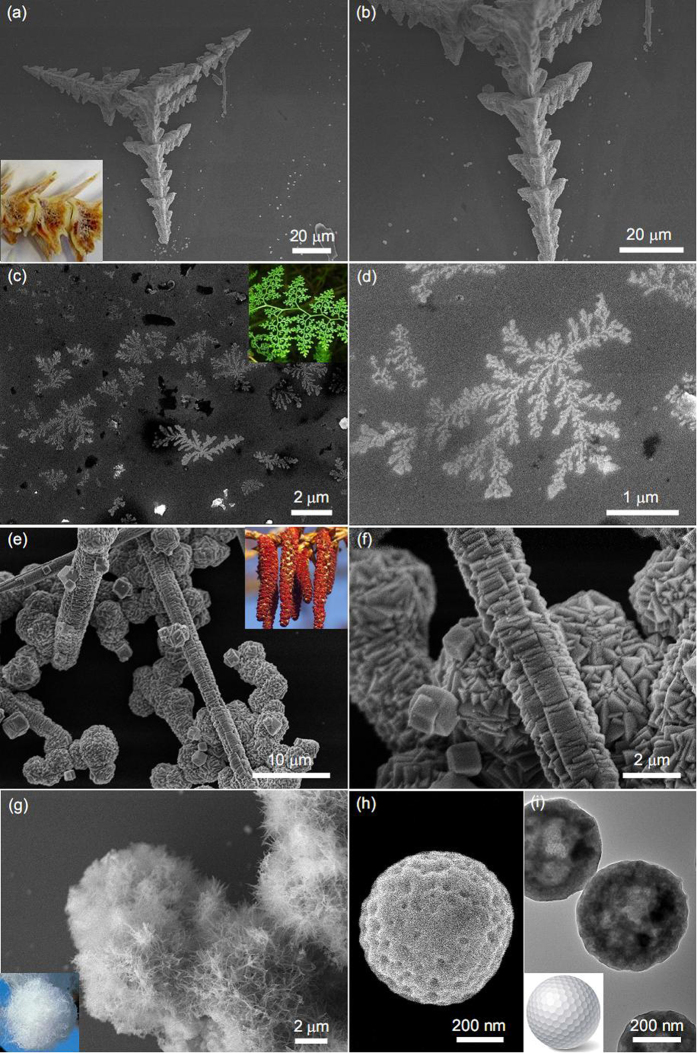
SEM images of the as-synthesized (**a,b**) fish bone-like, (**c,d**) pteridophyte-like, (**e,f**) poplar flower-like, and (**g**) cotton-like Cu@Cu_2_O products. (**h**) SEM and (**i**) TEM images of golfball-like Cu@ZnO products. Digital photographs of common fish bone, pteridophyte, poplar flower, cotton and golfball are shown as insets.

The chemical composition of the as-synthesized products are shown in Supplementary Figure S1, in which the XRD diffraction peaks of fish bone-like, pteridophyte-like, poplar flower-like, cotton-like architectures can be indexed to the cubic structure of Cu (JCPDS No. 85-1326) and Cu_2_O (JCPDS No. 78-2076), and the diffraction peaks of golfball-like architecture can be indexed to the cubic structure of Cu (JCPDS No. 85-1326) and hexagonal structure of ZnO (JCPDS No. 65-3411). The SEM images with low- and high- magnifications reveal the similarity of their morphologies to fish bone (Fig. 1a,b), pteridophyte (Fig. 1c,d), poplar flower (Fig. 1e,f), cotton (Fig. 1g) and golfball (Fig. 1h and TEM image in Fig. 1i) in our daily life.

The formation mechanism of these architectures mainly concerns three possible influencing factors. One is the reduction environment induced by the EG solvent, which is responsible for the mixed valence of nulvalent and monovalent copper ions in the final products. Secondly, the amount of reagent urea during fabrication of Cu@Cu_2_O products determines their geometry architectures. Urea not only can effectively prevent the aggregation of nanoparticles in the initial reaction stage, but also can kinetically control the growth rates of various crystallographic facets of Cu@Cu_2_O through selectively absorbing on these facets. If the amount of urea is high, the preformed nanoparticles tend to form nanowires to avoid aggregation to the greatest extent. The high dosage of urea also nonselectively covers nearly all crystallographic facets of Cu@Cu_2_O, and facilitates the longitudinal growth of these nanowires. After a long reaction time, the final product is of open-up network structure assembled by interconnected nanowires, e.g., the cotton-like architecture in this study. If the amount of urea is not that much, urea can effectively and selectively absorb on specific crystallographic facets of Cu@Cu_2_O, yielding secondary structures, e.g., fish bone-like and pteridophyte-like architectures depending on the reaction time. If the amount of urea is too low to cover all the preferential crystallographic facets of Cu@Cu_2_O, the secondary structure is runtish and lacks topological features, e.g., poplar flower-like architecture in this study. Lastly, the reaction temperature is not the least important in their morphology evolution. In comparison with the above low-temperature architectures, high temperature not only facilitates the formation of spherical structures to minimize the total Gibbs free energy, but also facilitates the fully transformation from Cu^2+^ ions to Cu in reduction environment because Cu^+^ions are unstable at high temperatures. This is the right case for our golfball-like Cu@ZnO architecture.

The TEM images of these architectures are separately shown in Supplementary Figures S2–S6. Obviously, their common feature lies in their rough surface texture which, upon closer scrutiny, reveals a disordered assembly of numerous nanoparticles. Regardless of their chemical composition, the selected area electron diffraction (SAED) patterns in Supplementary Figures S2–S6 show the coexistence of bright spots as well as dim aureoles, which are the salient features of single- and poly- crystals, respectively. These observations clearly demonstrate a blend of single- and poly- crystalline nature of these architectures. This unique crystalline characteristics can be further evidenced by the HRTEM images in Supplementary Figures S2–S6, in which (i) fringe spacings of ca. 2.08 and 1.81 Å can be assigned to (111) and (200) planes in copper phase, respectively; (ii) fringe spacings of ca. 2.46 and 2.98 Å correspond to (111) and (110) planes in Cu_2_O phase, respectively; and (iii) the fringe spacing of ca. 2.80 Å can be indexed to the (100) plane in ZnO phase. The coexistence of single- and poly- crystals induces a large quantity of defects (including vacancies and dislocations) and particle interfaces in these architectures, which will exert great influence on their magnetic properties. Supplementary Figure S2a shows the fish bone-like Cu@Cu_2_O product is composed of copious amount of nanoparticles, which align totally out of order and yield enormous interfaces in the material (Supplementary Figure S2c). As the reaction time amounts to 48h with other reaction conditions held constant (Supplementary Figure S3), the pteridophyte-like Cu@Cu_2_O product exhibits more distinct single-crystalline feature (Supplementary Figure S3b) with enhanced crystallinity in each composed nanoparticles (Supplementary Figure S3c). However, one can still observe conspicuous vacancies, dislocations and particle interfaces in this product (Supplementary Figure S3c). The poplar flower-like Cu@Cu_2_O product exhibits the best crystallinity among these architectures (Supplementary Figure S4b). This is presumably because the limited growth of secondary branch will facilitate the well crystallinity of the main trunk (Supplementary Figure S4c). In stark contrast, fringe spacings in the cotton-like Cu@Cu_2_O product are rather vague (Supplementary Figure S5c), in that the heavily capped urea inhibits its crystal growth. Similar weak crystallinity is also observed in the golfball-like Cu@ZnO product (Supplementary Figure S6b), in which the nucleation of Cu and ZnO components seems to dominate within the short reaction time of 30 min. Therefore, numerous defects and interfaces are clearly observed in Supplementary Figure S6c.

**Figure 2:**
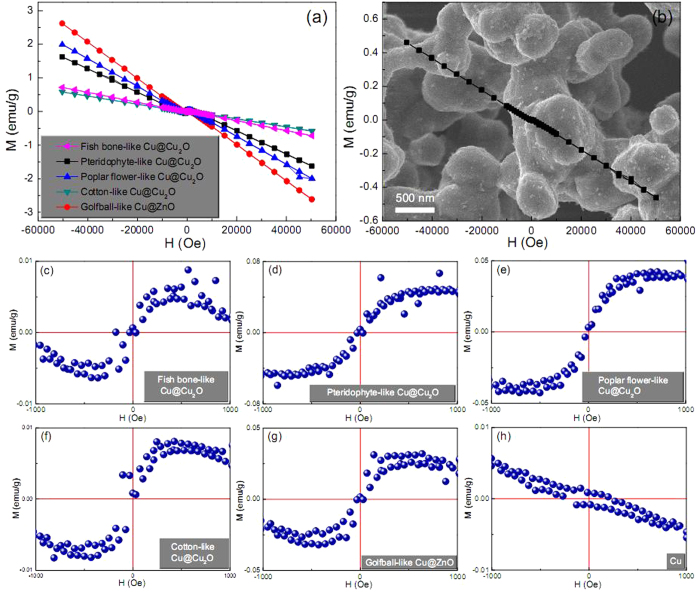
Magnetic hysteresis loops of the as-synthesized (**a**) Cu@Cu_2_O, Cu@ZnO products and (**b**) Cu reference sample measured at 300 K. The SEM image of Cu reference sample is shown as an inset in panel b. Low-field hysteresis loops of (**c**) fish bone-like, (**d**) pteridophyte-like, (**e**) poplar flower-like, (**f**) cotton-like, (**g**) golfball-like products and (**h**) Cu reference sample measured at 300 K.

Figure 2a shows room-temperature magnetic hysteresis loops of the as-synthesized Cu-based architectures. As a comparison, the hysteresis loop of pure Cu sample is used as a reference (Fig. 2b). Upon careful observation of the hysteresis loops in the field range from −1 to 1 kOe, the distinct ferromagnetic characteristics can be observed in fish bone-like (Fig. 2c), pteridophyte-like (Fig. 2d), poplar flower-like (Fig. 2e), cotton-like (Fig. 2f) Cu@Cu_2_O architectures and the golfball-like Cu@ZnO architecture (Fig. 2g); however, the Cu reference sample is of typical diamagnetic character (Fig. 2h). To be specific, clear technical magnetization process is evident on the low-field hysteresis loops for both Cu@Cu_2_O and Cu@ZnO architectures (Fig. 2c–g), although the high-field susceptibility after the technical saturation is negative (Fig. 2a). Note that these high-field susceptibility values differ significantly from that of pure Cu reference sample (Fig. 2b), indicating that the hysteresis loop of the conventional diamagnetic Cu is not simply superimposed over that of the ‘ferromagnetic phase’ in these architectures. Based on these observations, it is reasonable to speculate that at least part of the Cu phase may well alter its diamagnetic attribute and contribute to the ferromagnetic behaviors of these Cu-based architectures.

Such peculiar ferromagnetic properties of these architectures, assembled by non-ferromagnetic Cu and Cu_2_O or ZnO components, can be attributed to the presence of numerous defects and particle interfaces, as evidently shown in Supplementary Figures S2–S6. In the vicinity of these particle interfaces and defects, the local density of states at the Fermi level is greatly enhanced due to the reduced coordination number, and will promote the spontaneous magnetic moment within the small fraction of atoms nearby. In this scenario, the potential created by the mismatch of semiconductor and metal work functions will lead to a partial filling of the interface states^29^,^30^,^31^, and hence yielding a metallization of the interface^32^. In our case, conversion of Cu^0^ to Cu^+^ cations provides electrons, which can be diverted into the local defect density of states if it is energetically favorable. To be specific, if the energy gain from spin splitting exceeds the energy cost of the charge transfer, it is advantageous for the interface states around particle interfaces or defects to develop a magnetic moment, and order spontaneously provided there is a percolation path connecting them. That being the case, magnetic polarization in Cu_2_O or ZnO semiconductors induced by spin injection or charge transfer may extend for long distances, owing to low spin-orbit coupling and the absence of a hyperfine interaction^33^,^34^. In our system, the magnetic moments of atoms close to particle interfaces or defects order ferromagnetically, while the intact regions in particles remain diamagnetic, which makes up the mixture of ferromagnetic and diamagnetic characteristics in the hysteresis loops (Fig. 2).

Based on the above mechanism, the presence of charge-transfer process, induced by the defects and particle interfaces, is the root cause for the observed ferromagnetic behaviors. That means if the charge-transfer process is not available, the system will not exhibit ferromagnetic characteristics even though defects and particle interfaces are present. This point can be validated by the magnetic behaviors of pure Cu reference sample (Fig. 2b), which is of similar rough surface texture (inset of Fig. 2b) to Cu@Cu_2_O and Cu@ZnO architectures. The numerous particle interfaces or defects in this poly-crystalline Cu sample fail to invoke ferromagnetic responses and still maintain its typical diamagnetic attribute due to the absence of effective charge transfer. This charge transfer process mainly involves the motion of electrons moving from metal Cu to the crystal lattice of Cu_2_O or ZnO. Suchlike charge transport can be described by small polaron model, in which an electron (polaron) induces a localized distortion on its lattice surroundings due to its accompanying polarizing field^35^,^36^. The Cu^+^ and Zn^2+^ cations on the surface of Cu_2_O and ZnO components are reduced into lower ionic states Cu^(1−*x*)+^ and Zn^(2−*y*)+^ (0 < *x* < 1, 0 < *y* < 2), respectively, by taking up electrons from neighboring Cu atoms on the surface of metal Cu particles, which are synchronously oxidized into Cu^*x*+^ and Cu^*y*+^ cations, respectively. This will result in Cu^(1−*x*)+^-Cu^*x*+^-O and Zn^(2−*y*)+^-Cu^*y*+^-O interfacial layers where the electron charge transfer is likely to take place, and these interfacial layers are essential to the observed ferromagnetism in these Cu@Cu_2_O and Cu@ZnO architectures.

**Figure 3:**
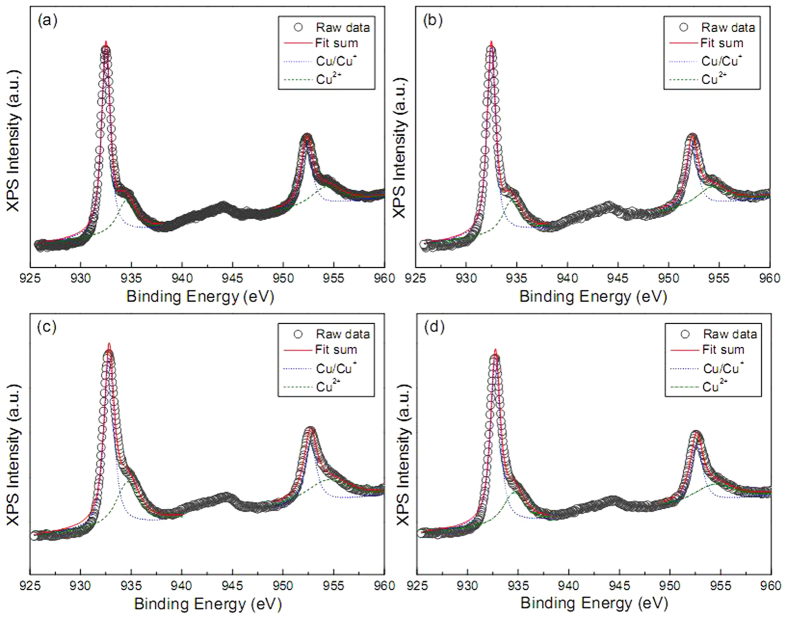
XPS high-resolution scan of Cu 2p_3/2_ and 2p_1/2_ peaks measured on (**a**) fish bone-like, (**b**) pteridophyte-like, (**c**) poplar flower-like, and (**d**) cotton-like Cu@Cu_2_O products.

**Figure 4:**
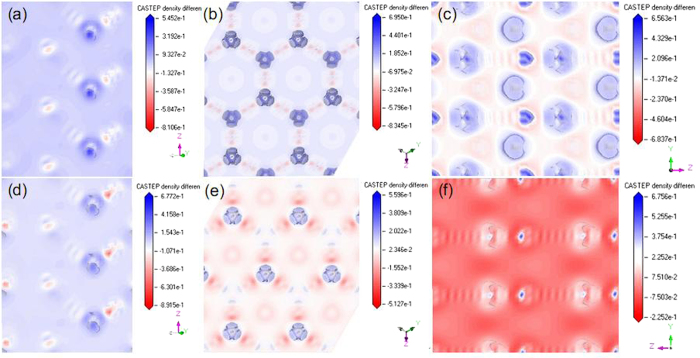
Calculated charge density difference map for (**a**) Cu_2_O(110), (**b**) Cu_2_O(111), (**c**) ZnO(100) surfaces, and (**d**) Cu(111)/Cu_2_O(110), (**e**) Cu(200)/Cu_2_O(111), (**f**) Cu(111)/ZnO(100) interfaces. Blue and red regions correspond to the zones of electron gains and losses, respectively.

The XPS survey may evidence the alteration of copper state. Figure 3 shows the Cu 2p core-level XPS spectra of these Cu@Cu_2_O products. The Cu 2p_3/2_ and 2p_1/2_ core-levels are located at ca. 932.77 and ca. 952.77 eV, respectively. The broad satellite peaks centered on ca. 943 eV is characteristic of Cu^2+^ species, which originate from the inevitable oxidation of Cu/Cu^+^ species in our product. Because the above XPS characterization is unable to provide definitive evidence on the charge transfer process, first principle calculations were carried out by using the Cambridge sequential total energy package (CASTEP)^37^,^38^. The Perdew-Burke-Ernzerhof function^39^,^40^ within the generalized gradient approximation (GGA-PBE)^41^ was used. An on-site Coulomb correction was applied to the Local Density Approximation (LDA + U) with U = 5 eV^42^. Ionic cores were described by the ultrasoft pseudopotential^43^, and the Kohn-Sham one-electron states were expanded in a plane wave basis set up to 340 eV (cut-off energy). Brillouin zone integration was approximated by a sum over special *k* points chosen using the Monhorst-Pack scheme^44^. The convergence criteria for the structure optimization were set to (a) an SCF tolerance of 1.0 × 10^−6^ eV/atom, (b) an energy tolerance of 1.0 × 10^−5^ eV/atom, (c) a maximum force tolerance of 0.03 eV/Å, and (d) a maximum displacement tolerance of 1.0 × 10^−4^ nm. Our calculations focus on three typical interfaces, including Cu(111)/Cu_2_O(110), Cu(200)/Cu_2_O(111) and Cu(111)/ZnO(100), which are quite common in these architectures as shown in Supplementary Figures S2–S6. Figure 4 illustrates the charge density difference map at above three interfaces. By comparing them with the charge density difference map at Cu_2_O(110), Cu_2_O(111) and ZnO(100) reference planes, it is apparent that Cu(111)/Cu_2_O(110), Cu(200)/Cu_2_O(111) and Cu(111)/ZnO(100) interfaces possess greater electron density. The remarkable delocalization of electrons at these three interfaces verify the great electronic capacity losses from neighboring Cu(111) and Cu(200) planes, namely, charge transfer process. Most notably, Cu(111)/ZnO(100) interface exhibits the largest overlapping probability of electron clouds, which even smears the feature of electron gains at oxygen sites. This phenomenon may be attributed to more electrons are transferred from metal Cu to Zn^2+^ cations in Cu@ZnO architecture than to Cu^+^ cations in Cu@Cu_2_O architecture.

**Figure 5:**
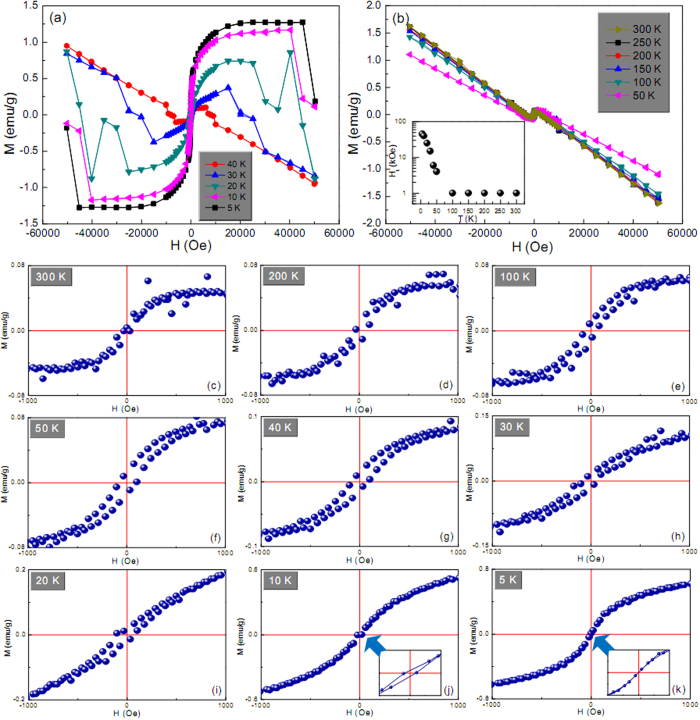
(**a,b**) Magnetic hysteresis loops of the pteridophyte-like Cu@Cu_2_O product measured at different temperatures. Temperature dependence of *H*_t_ values is shown as an inset in panel b. Low-field hysteresis loops taken from panel a and b at (**c**) 300, (**d**) 200, (**e**) 100, (**f**) 50, (**g**) 40, (**h**) 30, (**i**) 20, (**j**) 10, and (**k**) 5 K.

**Figure 6:**
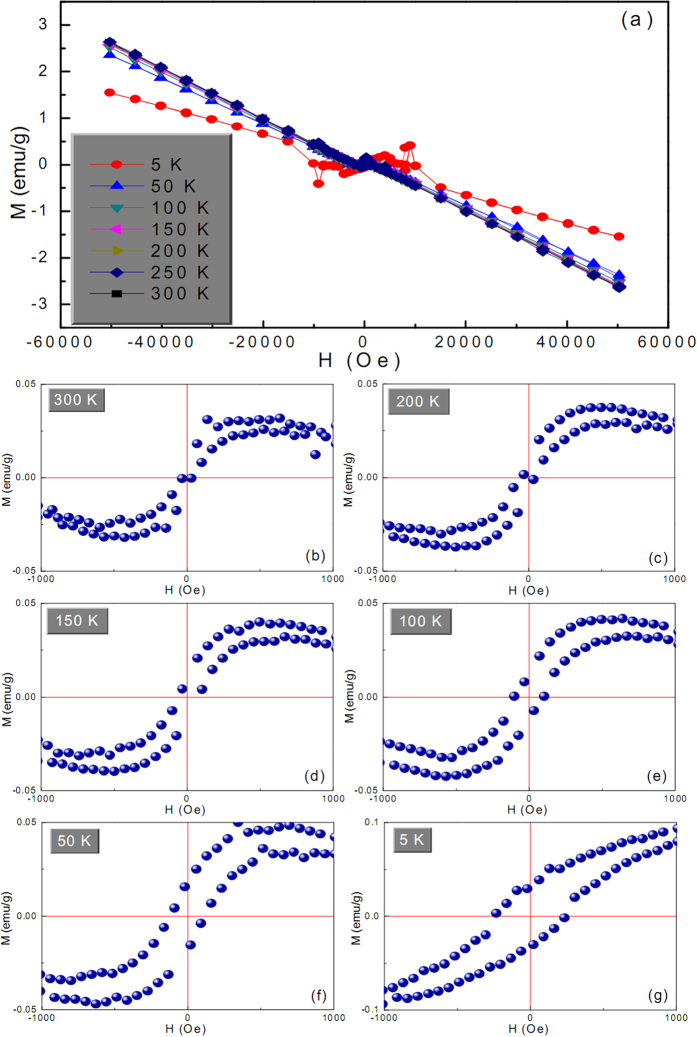
(**a**) Magnetic hysteresis loops of the golfball-like Cu@ZnO product measured at different temperatures. Low-field hysteresis loops taken from panel a at (**b**) 300, (**c**) 200, (**d**) 150, (**e**) 100, (**f**) 50, and (**g**) 5 K.

**Figure 7:**
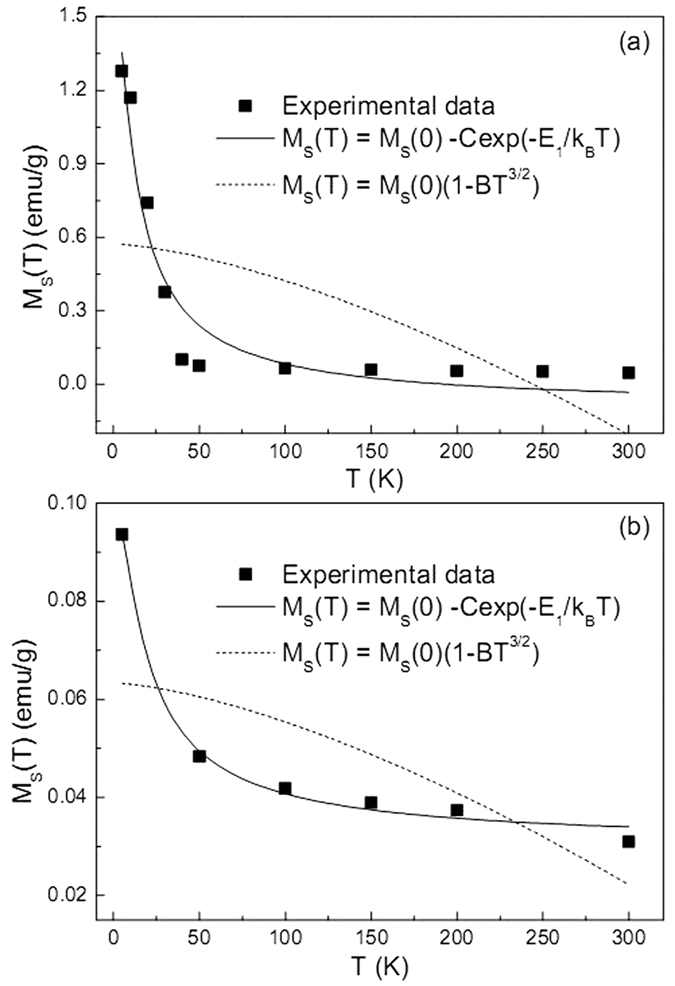
Temperature dependence of *M*_*S*_(*T*) values of (**a**) pteridophyte-like Cu@Cu_2_O and (**b**) golfball-like Cu@ZnO products.

The different amount of transferred electrons through interfaces and defects may have profound effect on the expected ferromagnetism. To verify this point, the magnetic properties of pteridophyte-like Cu@Cu_2_O and golfball-like Cu@ZnO architectures, which are of different electron density at interfaces (Fig. 4), are thoroughly investigated. Figure 5a,b show that the pteridophyte-like Cu@Cu_2_O architecture exhibits conspicuous ferromagnetism in varying field ranges depending on temperature. Beyond this field range, it shows a typical diamagnetic feature due to the presence of diamagnetic phase. To exclude the possibility of SQUID stability problem at high magnetic fields, the sample was re-measured in another SQUID magnetometry (MPMS-7XL) below 40 K. The obtained results, as shown in Supplementary Figure S7, bear striking similarities with those in Fig. 5a, indicating the hopping data are not artifacts of the experiment or attributed to inadequate statistics while averaging over a macroscopic sample. The critical transition field from ferromagnetism to diamagnetism is denoted as *H*_t_. The inset of Fig. 5b clearly shows a monotonically decreasing tendency of *H*_t_ values as increasing the ambient temperature. The origin and variation tendency of these *H*_t_ values need further study. Moreover, the hysteresis loop in Fig. 5j,k nearly passes across the origin (inset of Fig. 5j,k). Seemingly, this is a transition state between ferromagnetism and diamagnetism. Therefore, it follows that the transition from ferromagnetism to diamagnetism not only happens above some critical fields to exhibit data-hopping phenomena, but also occurs below some critical temperatures to exhibit superparamagnetic-like behavior. By contrast, golfball-like Cu@ZnO behaves as a ferromagnet merely within a narrower field range of −1 to 1 kOe (Fig. 6a), which is also much narrower than that in defect-induced ferromagnetic ZnO^27^. But more strikingly, the magnetization at ±1 kOe of golfball-like Cu@ZnO product is about 0.09 and 0.03 emu/g at 5 and 300 K, respectively. These magnetization values are much larger than the reported values (<0.02 emu/g) in defect-induced ferromagnetic ZnO^27^. More importantly, the coercivity of golfball-like Cu@ZnO product at 5 K amounts to 250 Oe, which is also much higher than the reported value of ca. 80 Oe in defect-induced ferromagnetic ZnO^27^. Thus, it can be inferred that with Cu charge transfer, the ferromagnetism is quite distinctive from the case without charge transfer. In addition, despite of lower coercity values of pteridophyte-like Cu@Cu_2_O architecture, it possesses higher saturation magnetization (*M*_*S*_) values (Fig. 5c–k) than those of golfball-like Cu@ZnO architecture (Fig. 6b–g) at any given temperature. The temperature-dependent *M*_*S*_ values [*M*_*S*_(*T*)] of these two architectures are shown in Fig. 7. Obviously, the *M*_*S*_(*T*) values of pteridophyte-like Cu@Cu_2_O architecture are about an order of magnitude larger than those of golfball-like Cu@ZnO architecture.

Generally, the demagnetization of ferromagnetic materials at temperatures much below the Curie temperature is due to the excitation of long-wavelength spin waves whose energy is characterized by the spin-wave stiffness coefficient *D*. In case of a continuous distribution of spin-wave states in the bulk, the temperature dependence of *M*_*S*_ values can be given by the Bloch law (equation (1))


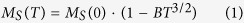


where *B* = 2.6149 *V*_0_ (*k*_B_/4π*D*)^3/2^, *V*_0_ is the atomic volume. But in nano-sized particles, the thermal dependence of *M*_*S*_ values deviates from the expected Bloch law as the magnons with wavelength larger than the particle dimensions cannot be excited and a threshold of thermal energy is required to generate spin waves^45^. The finite size of the particles led to a discrete set of energy values corresponding to a discrete spectrum of spin-wave modes. At temperatures well below the Curie temperature of the material, *M*_S_ can be approximately written as^46^


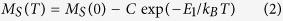


neglecting the higher-order terms. Here, *E*_1_ is the excitation energy of spin wave in the limit of small wave vectors (*k*_1_ → 0), i.e., *E*_1_ = *Dk*_1_^2^. Equations (1) and (2) are both utilized to fit the temperature-dependent *M*_*S*_ values in our experiments (Fig. 7). The fitted parameters are summarized and listed in Supplementary Table S1. It is evident that, whether for the case of pteridophyte-like Cu@Cu_2_O or golfball-like Cu@ZnO architecture, the *M*_*S*_(*T*) data can be well fitted by equation (2). This means the observed ferromagnetism in these two architectures are totally contributed by nano-sized particles. This finding is in excellent consistency with our aforementioned ferromagnetic mechanism, in which particle interfaces and defects are considered to be actually responsible for such ferromagnetism. Notably, the *E*_1_ value of pteridophyte-like Cu@Cu_2_O (ca. 1.011 meV) is appreciably lower than that of the golfball-like Cu@ZnO (ca. 1.502 meV), indicating that the spin wave in pteridophyte-like Cu@Cu_2_O architecture is much easier to be excited, and hence contributes to its stronger ferromagnetism, as observed in Fig. 5a.

**Figure 8:**
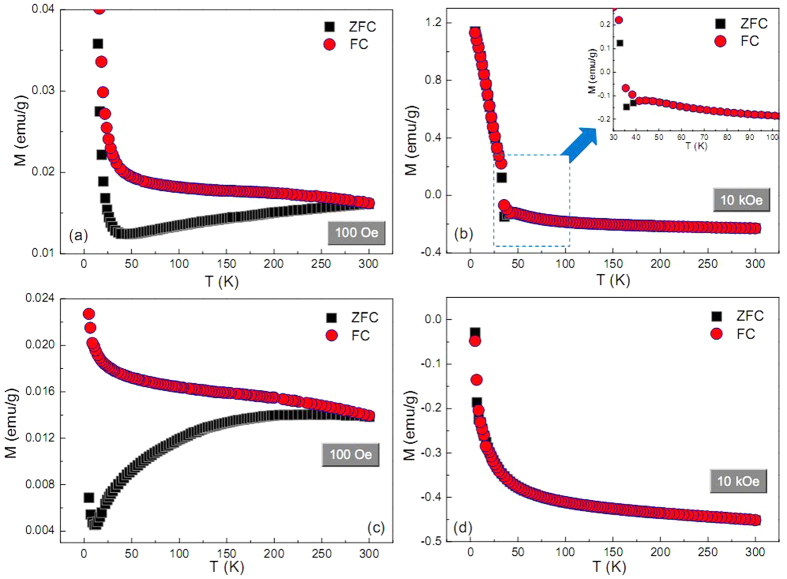
*M*_ZFC_(*T*) and *M*_FC_(*T*) curves measured at (**a,c**) 100 and (**b,d**) 10000 Oe for the (**a,b**) pteridophyte-like Cu@Cu_2_O and (**c,d**) golfball-like Cu@ZnO products. Inset: temperature-dependent magnetization curves depicted in low temperature range.

To seek deep insight into the magnetic interactions among Cu/Cu_2_O or Cu/ZnO ferromagnetic units, temperature dependent zero field-cooled (ZFC) and field cooled (FC) magnetization values [denoted as *M*_ZFC_(*T*) and *M*_FC_(*T*), respectively] were measured in the field of 100 and 10000 Oe separately (Fig. 8). In the field of 100 Oe for pteridophyte-like Cu@Cu_2_O and golfball-like Cu@ZnO architectures, the *M*_ZFC_(*T*) and *M*_FC_(*T*) curves do not overlap over the whole range of temperatures up to 300 K (Fig. 8a,c). This substantial deviation indicates that the Cu/Cu_2_O or Cu/ZnO ferromagnetic units are not isolated but are instead strongly coupled by magnetic dipolar interactions^47^,^48^. Such dipolar interactions are mainly attributed to the labyrinthine particle interfaces, which are the origins to yield ferromagnetism in these architectures. In the field of 10 kOe, the *M*_ZFC_(*T*) and *M*_FC_(*T*) curves of golfball-like Cu@ZnO architecture exclusively overlap over the whole range of temperatures up to 300 K and exhibit typical diamagnetic feature because the applied field of 10 kOe is much above its ferromagnetic field range of −1 ~ +1 kOe (Fig. 6b–g). This, however, is not the case for the pteridophyte-like Cu@Cu_2_O architecture under the field of 10 kOe. Figure 8b shows its *M*_ZFC_(*T*) and *M*_FC_(*T*) curves virtually overlap in the whole temperature range except for 30~40 K (inset of Fig. 8b), and do not reveal any sign of diamagnetic characteristics. Proverbially, FC magnetization measurements alone can provide information about magnetic interactions between nanoparticles. In many types of magnetic systems, the FC magnetization increases with decreasing temperature due to the decreased thermal fluctuations and spins aligning with the applied field^49^. Dipolar interaction between particles suppresses these fluctuations, and thus the increase in magnetization during a FC experiment is inversely proportional to the amount of interaction^50^. The *M*_FC_(*T*) curve of pteridophyte-like Cu@Cu_2_O architecture in Fig. 8b shows a steady plateau with lowering the temperature until 45 K, followed by a steep increase at lower temperatures. This behavior is in accordance with the presence of strong magnetic interactions above 45 K^51^,^52^. Strong dipolar interactions block the rotation of spins to align with the field, which results in the virtually unchanged FC magnetization.

## Conclusions

In summary, fish bone-like, pteridophyte-like, poplar flower-like, cotton-like Cu@Cu_2_O architectures and golfball-like Cu@ZnO architecture were successfully fabricated by a versatile solution route. These architectures exhibited peculiar room-temperature ferromagnetism, which was derived from the charge transfer processes from metal Cu to semiconductors Cu_2_O and ZnO. First principle calculations were then carried out to verify these charge transfer processes, and the small polaron model was utilized to explain their ferromagnetic origin. By investigating the ferromagnetism of pteridophyte-like Cu@Cu_2_O and golfball-like Cu@ZnO architectures, it was found that different metallization at metal-semiconductor interfaces would induce different ferromagnetic properties, including coercivity, saturation magnetization and magnetic interactions.

## Methods

All chemicals were analytical grade and used as received without further purification.

### Syntheses of Cu@Cu_2_O architectures

In typical syntheses, Cu(NO_3_)_2_·3H_2_O and urea with a molar ratio of 2:1, 1:1 and 1:3 were dissolved and stirred in 50 mL of ethylene glycol (EG) at room temperature until a clarified blue solution was formed. Then the mixture was transferred into a Teflon-lined stainless-steel autoclave with a capacity of 100 mL for solvothermal treatment at different temperatures for different time. The as-obtained precipitates were repeatedly washed with deionized water and ethanol, and finally dried at 60 °C for 4h. To obtain fish bone-like and cotton-like Cu@Cu_2_O architectures, the molar ratio of Cu(NO_3_)_2_·3H_2_O and urea were set to be 1:1 and 1:3, respectively, and their solvothermal reactions were both conducted at 140 °C for 12 h. To obtain pteridophyte-like and poplar flower-like Cu@Cu_2_O architectures, the molar ratio of Cu(NO_3_)_2_·3H_2_O and urea were set to be 1:1 and 2:1, respectively, and their solvothermal reactions were both conducted at 140 °C for 48 h.

### Synthesis of golfball-like Cu@ZnO architecture

In a typical synthesis, an equimolar (0.625 mmol) mixture of Cu(NO_3_)_2_·3H_2_O and Zn(NO_3_)_2_·6H_2_O was dissolved and stirred in 25 mL of EG at room temperature until a homogeneous solution was formed. The solution was subsequently heated and refluxed at 200 °C for 30 min, during which the precipitate could be visualized. The precipitate was collected by centrifugation and rinsed with deionized water and ethanol, and finally dried at 60 °C for 4 h.

### Synthesis of Cu reference sample

In a typical synthesis, 5 mmol of CuCl_2_·2H_2_O and 1 g of polyvinyl pyrrolidone (PVP, M = 10000) were dissolved and stirred in 40 mL of deionized water at room temperature until a homogeneous solution was formed. Then 10 mL of N_2_H_4_·H_2_O (80%) was added dropwise. After that, the solution was transferred into a Teflon-lined stainless-steel autoclave with a capacity of 100 mL for hydrothermal treatment at 180 °C for 24 h. The as-obtained precipitate was repeatedly washed with deionized water and ethanol, and finally dried at 60 °C for 4 h.

XRD patterns were recorded on a powder X-ray diffractometer (Rigaku D/max-rA) equipped with a rotating anode and a Cu -K_α1_ radiation source (λ = 1.5406 Å) at a step width of 0.02°. Scanning electron microscope (SEM) images were collected on a field -emission scanning electron microscope (JEOL JSM-6700F). Transmission electron microscopy (TEM) was performed on the JEOL 2010 TEM with an operating voltage of 200 kV. The high-resolution electron microscopy (HRTEM) experiments were conducted using a Field Emission Gun (FEG) JEOL 2010F microscope with a point resolution of 0.19 nm. X-ray photoelectron spectroscopy (XPS) was performed on VG ESCALAB 220i-XL system equipped with a monochromatic X-ray source in an ultra-high-vacuum chamber at a pressure lower than 1.0 × 10^−9^ Torr. Peak positions are referenced to the adventitious C 1s peak taken to be 284.8 eV. The magnetic measurements of powder samples were conducted on superconducting quantum interference device (SQUID) magnetometry (MPMS-5XL, Quantum Design).

## Additional Information

**How to cite this article**: Ma, J. *et al*. Assembling non-ferromagnetic materials to ferromagnetic architectures using metal-semiconductor interfaces. *Sci. Rep.* **6**, 34404; doi: 10.1038/srep34404 (2016).

## Supplementary Material

Supplementary Information
