# Supplementary material for: Assembling non-ferromagnetic materials to ferromagnetic architectures using metal-semiconductor interfaces
Source: Sci Rep. 2016 Sep 29;6:34404. doi: 10.1038/srep34404 (PMC5041146; doi:10.1038/srep34404)
Supplement: Supplementary Information [file srep34404-s1.pdf]

Supplementary Information

Assembling non-ferromagnetic materials to ferromagnetic architectures using metal-semiconductor interfaces

*Ji Ma, Chunting Liu, and Kezheng Chen**

Lab of Functional and Biomedical Nanomaterials, College of Materials Science and Engineering, Qingdao University of Science and Technology, Qingdao 266042, China.

* To whom correspondence should be addressed. Tel: +86-532-84022509. Fax: +86-532-84022509. E-Mail: kchen@qust.edu.cn

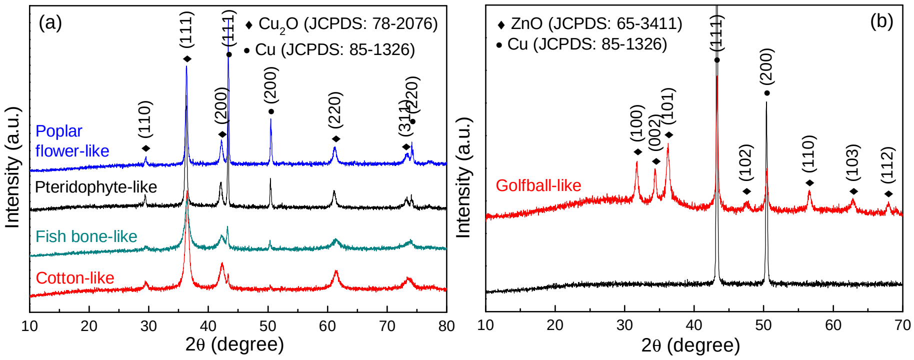

Figure S1 XRD patterns of the as-synthesized (a) Cu@Cu_2O, and (b) Cu@ZnO and Cu products.

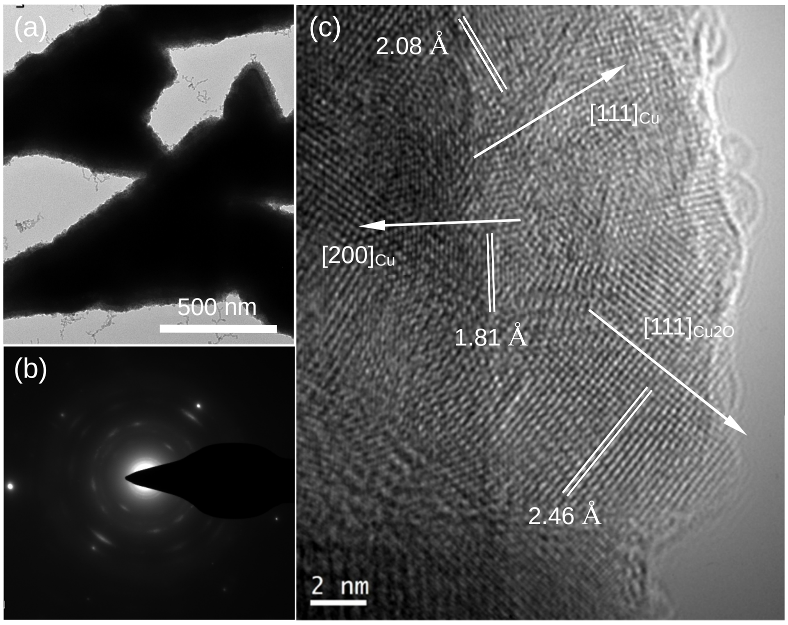

Figure S2 (a) TEM image, (b) SAED pattern and (c) HRTEM image of fish bone-like Cu@Cu_2O product.

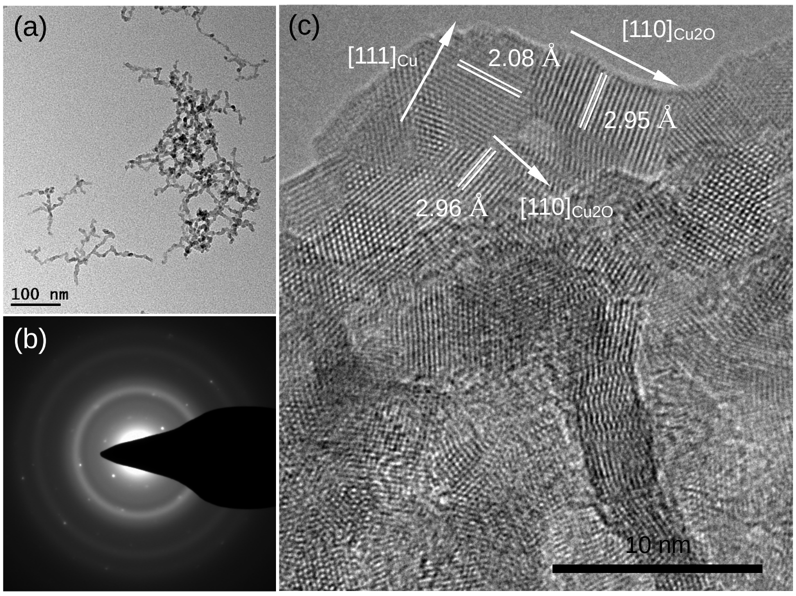

Figure S3 (a) TEM image, (b) SAED pattern and (c) HRTEM image of pteridophyte-like Cu@Cu_2O product.

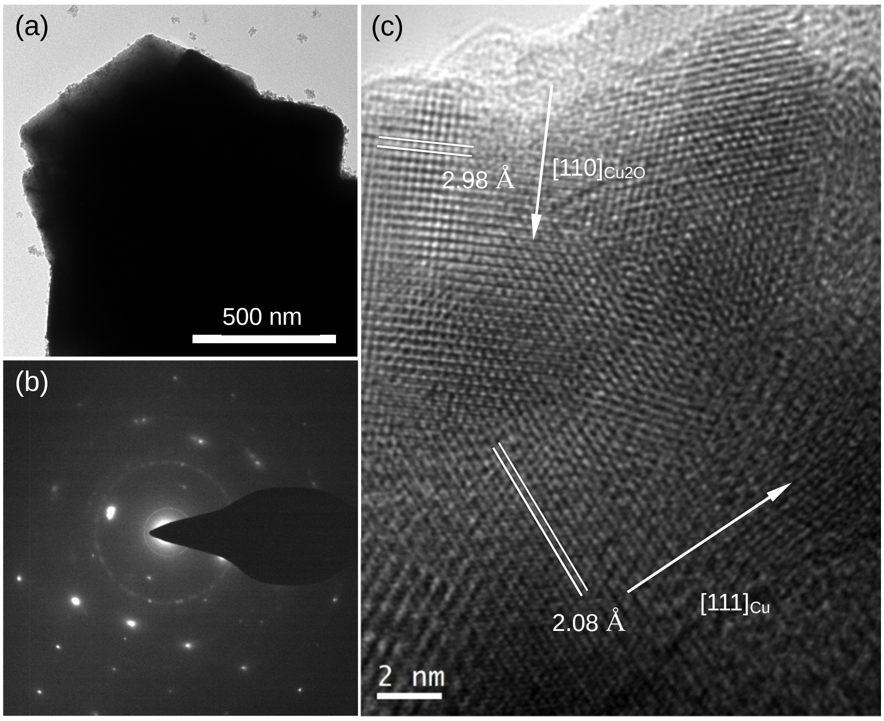

Figure S4 (a) TEM image, (b) SAED pattern and (c) HRTEM image of poplar flower-like Cu@Cu_2O product.

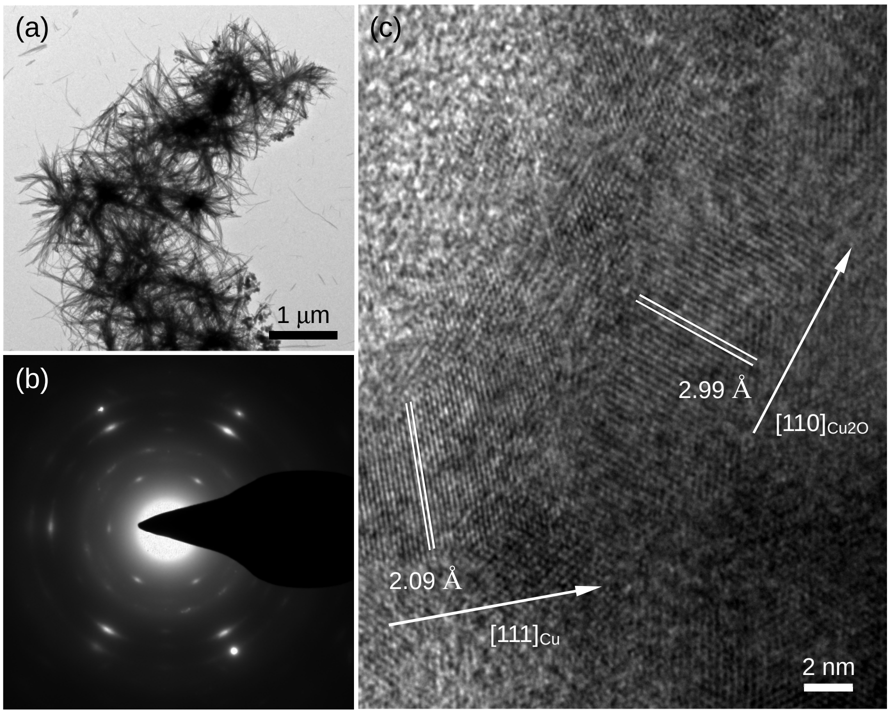

Figure S5 (a) TEM image, (b) SAED pattern and (c) HRTEM image of cotton-like $Cu@Cu_2O$ product.

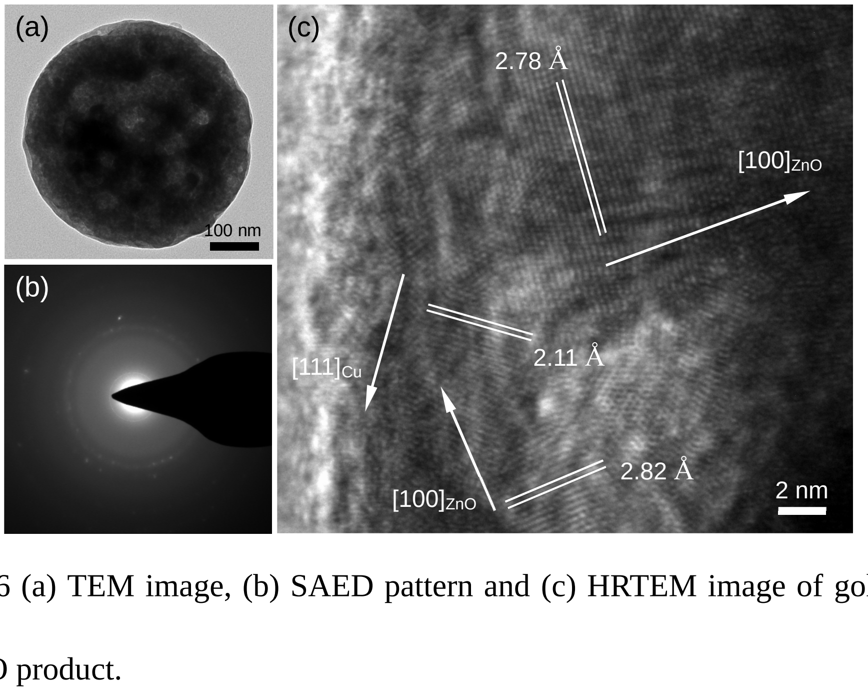

Figure S6 (a) TEM image, (b) SAED pattern and (c) HRTEM image of golfball-like Cu@ZnO product.

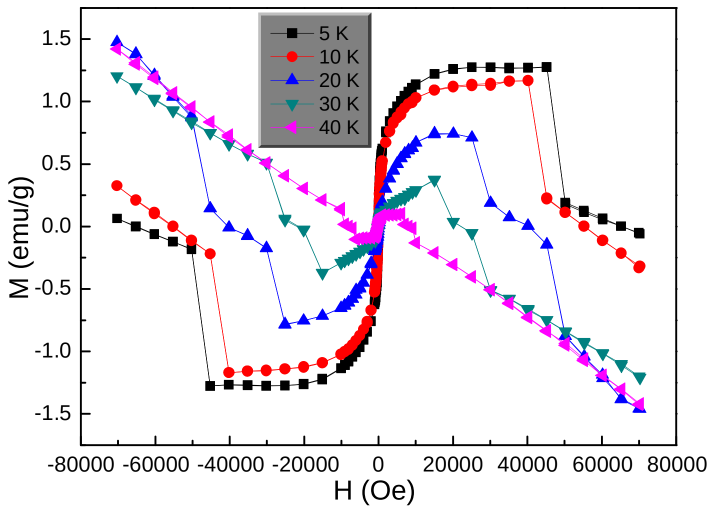

Figure S7 Magnetic hysteresis loops of the pteridophyte-like $Cu@Cu_2O$ product measured at different temperatures.

Table S1 Fitted parameters according to equation (2) for pteridophyte-like $Cu@Cu_2O$ and golfball-like Cu@ZnO products.

Samples	$M_S(0)$ (emu/g)	C (emu/g)	E_1 (meV)	R^2
Pteridophyte-like $Cu@Cu_2O$	1.507±0.245	1.601±0.225	1.011±0.351	0.924
Golfball-like Cu@ZnO	0.096±0.003	0.065±0.003	1.502±0.363	0.989
